# Comparison of Postoperative Outcomes Between Open Reduction and Internal Fixation and Ilizarov for Schatzker Type V and Type VI Fractures

**DOI:** 10.7759/cureus.4902

**Published:** 2019-06-14

**Authors:** Muhammad Tahir, Sandeep Kumar, Saeed A Shaikh, Allah Rakhio Jamali

**Affiliations:** 1 Orthopaedics, Jinnah Postgraduate Medical Center, Karachi, PAK

**Keywords:** tibial plateau, ilizarov, open reduction and internal fixation (orif), fractures

## Abstract

Introduction

Open reduction and internal fixation (ORIF), hybrid fixation, and external fixation are available treatment options for bicondylar fractures of the proximal tibia but which one is superior to the others is not yet established. Therefore, the study aimed to establish a gold standard treatment option for bicondylar fractures by comparing the clinical, functional, and radiological outcomes managed by Ilizarov and ORIF at 24 months.

Methods

This was a retrospective study conducted from 2009 to 2014 at a public sector, tertiary care, level I trauma center. Patients with Schatzker type V and type VI open and closed fractures were included. Floating knee, pathological fractures of the tibia, and patients having medical conditions were excluded from the study. Honkonen and Jarvinen (HJ) criteria for subjective, clinical, radiological, and functional outcomes were used to compare between the two groups at 24 months. Demographic data included age, gender, Schatzker type, mechanism of injury, and range of knee flexion. Chi-square was used to find the level of significance, which was 0.05.

Results

A total of 137 patients were included in this study, with 68 patients in the ORIF group and 69 in the Ilizarov group during the study period. The mean age of the patients was 45.08 ± 10.52, respectively. The male to female distribution was 107/30 (78.1% and 21.89%). According to the mechanism of injury, road traffic accidents (RTA) were the primary cause of injury: 96 (70.07%), falls were 21 (15.32%), and gunshots were 18 (13.13%). Seventy-four were Schatzker type VI (54.01%) whereas 63 (45.98) were Shcatzker V. The average knee flexion at 24 months was 115.51 ± 16.82. There were no differences in the clinical, functional, and radiological outcomes at 24 months between the two treatment groups.

Conclusion

No single treatment option can be applied in all cases, and the decision depends on the complexity of the injury, the surgeon’s expertise, and host factors.

## Introduction

Schatzker type V and type VI fractures, also known as bicondylar fractures of the proximal tibia, are complex and challenging injuries that occur due to high-energy trauma, often associated with extensive soft tissue injuries [1]. The severity of the injury (degree of comminution), clinical presentation, bone quality, host-related factors, and functional status often dictate the choice of treatment [2].

Schatzker Type V and Type VI fractures mostly require surgical management, with the main aim of restoring the articular congruity and attaining anatomical reduction and stable fracture fixation adequate for early/immediate knee motion [3]. The various surgical techniques available are open reduction and internal fixation (ORIF) with osteosynthesis and Ilizarov [4-6]. Treatment aims to obtain a stable, aligned, mobile, and painless joint and prevent post-traumatic osteoarthritis [7].

The standard method treatment is ORIF with osteosynthesis and dual plating is the most stable mechanical construct in such fractures [8]. The benefits with this approach are the optimal visualization of the fracture for reduction and fixation, absolute stability, and restoration of joint congruity. The drawbacks of this method are extensive soft-tissue dissection over the subcutaneous proximal end of the tibia, excessive periosteal stripping, and devascularization of the periosteum, skin necrosis, and infection [9-11].

Recently, the less invasive stabilization system (LISS) plating system and Ilizarov have been advocated to minimize ORIF complications. According to the Canadian Orthopaedic Trauma Society, the benefits of Ilizarov are that it can be used in open fractures, whereas ORIF is limited to closed fractures. Another advantage of Ilizarov is that it does not disrupt the blood supply, and thus the periosteal supply remains intact, it allows early weight bearing and thus leads to a shorter hospital stay [12]. Another advantage of Ilizarov is that it is applied percutaneously. Therefore, the apparatus does not disturb fracture biology and provides physiologic fixation [13-14]. Furthermore, there is minimal soft tissue disruption, and it allows correction in multiple planes [15].

The significant pitfalls of Ilizarov are pin-site infections and patient compliance with the cumbersome apparatus, the necessity of strict follow-up, and joint congruity is not restored always [14].

Overall, there is still ambiguity over which surgical method is superior to the other, and there is no defined gold standard technique for these fractures. Therefore, the study aimed to compare the functional, radiological, and clinical outcomes of Schatzaker type V and VI fractures treated by Ilizarov versus internal fixation and reached a conclusion on which treatment modality was better than the other.

## Materials and methods

Study demographics

With the approval of the Medical Ethics Review Board Committee, the study was conducted in a two-unit, 80-bedded department of trauma and orthopedics of a level-I, tertiary care public sector hospital from 2009-2014.

Study design

The study was designed as a retrospective case-control study to compare the subjective, clinical, radiological, and functional outcomes of patients undergoing treatment for bicondylar fractures.

Study population

The study enrolled all the patients that were treated between 2009 and 2014 in both units.

Study protocol

The protocol included accessing the patient records retrospectively from the Hospital Internal Management System (HIMS). Patients were divided into two groups. Controls were labeled as group A (ORIF with dual plating), whereas the cases were designated as group B (Ilizarov). Patients were assigned to one of two treatment groups following simple randomization procedures (computerized random numbers).

Inclusion and exclusion criteria

**Figure 1:**
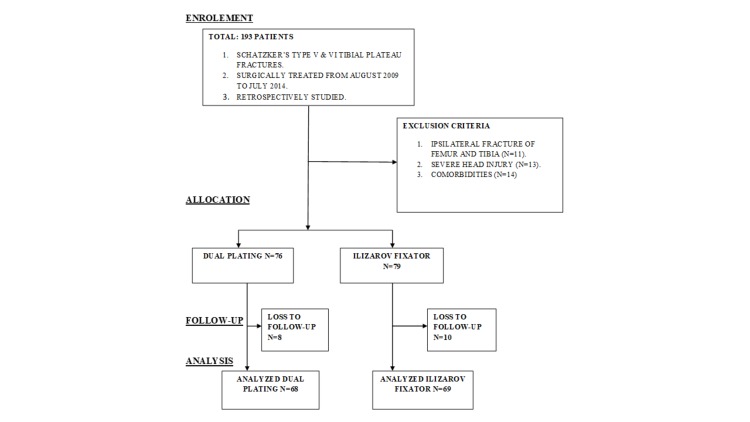
Diagram representing the recruitment process

Patients having an isolated open or closed Schatzker V or Schatzker VI were included in the study. Pathological fracture of the tibia, floating knee, polytrauma, patients lost to follow-up before 24 months, and significant comorbidities like congestive heart failure, hypertension, chronic liver disease, stroke, or obstructive lung disease were excluded from the study. Figure 1 shows the recruitment process.

Final outcome

The outcome to be assessed was the clinical, radiological, and functional outcomes and a comparison of the results of both treatment options. Postoperative outcomes were analyzed and documented at 24 months from the date of surgery using the Honkonen and Jarvinen (HJ) criteria.

Tool for assessment

In 1992, Honkonen and Jarvinen et al. described a comprehensive grading system according to which tibial plateau fractures would be classified as excellent, good, fair, or poor [16].

The HJ criteria are based upon four parameters: subjective, clinical assessment, functional evaluation, and radiological scoring. The subjective assessment consists of the frequency of symptoms experienced by the patient: daily, weekly, fortnightly, monthly, or never. The clinical evaluation is based upon extension lag, range of flexion, and thigh atrophy. The functional assessment comprises the ability to walk, climb stairs, jump, squat, and duck walk. The radiological evaluation includes the degree of varus/valgus and tilting of the plateau, articular step-off and condylar widening in millimeters, and the relative joint space narrowing indicates degenerative changes after the plateau fracture.

Statistical analysis

The analysis was conducted on SPSS version 22 (IBM Corp., Armonk, NY, US). Mean and standard deviation were calculated for quantitative data. Frequency and percentages were calculated for qualitative data. Groups A and B were compared with each other for postoperative HJ outcomes by using the chi-square test, taking p-value ≤ 0.05 as significant.

## Results

A total of 137 patients were included in this study, with 68 patients in the ORIF group and 69 in the Ilizarov group during the study period. The mean age of the patients was 45.08 ± 10.52, respectively. The male to female distribution was 107/30 (78.1% and 21.89%). According to the mechanism of injury, road traffic accidents (RTA) were the primary cause of injury 96 (70.07%), falls were 21 (15.32%), and gunshots were 18 (13.13%). Seventy-four were Schatzker type VI (54.01%) whereas 63 (45.98) were Shcatzker V. The average knee flexion at 24 months was 115.51 ± 16.82. Table 1 compares the demographics of both groups.

**Table 1:** Demographics of ORIF and Ilizarov groups Data in bracket indicate the standard deviation for continuous variables, whereas the values in brackets for categorical variable shows percentages. ORIF - Open Reduction Internal Fixation

VARIABLES	ORIF	ILIZAROV
Age (Years)	40.01 (±11.07)	50.07 (±7.05)
Knee Flexion (Degrees)	111.71 (± 17.51)	119.26 (±15.32)
Males	59 (86.8%)	48 (69.6%)
Females	9 (13.2%)	21 (30.4%)
Road Traffic Accidents	52 (76.47%)	46 (66.71%)
Fall	10 (14.70%)	11 (15.94%)
Gunshot	6 (8.82%)	12 (17.39%)
Schatzker V	37 (54.41%)	26 (37.68%)
Schatzker VI	31 (45.58%)	43 (62.31%)

There was no significance established as to which treatment option was superior to the other in terms of subjective, clinical, radiological, and functional outcomes at 24 months, as p-value was higher than 0.05. Table 2 illustrates the clinical, functional, and radiological outcome according to the Honkonen and Jarvinen criteria and Figure 1 shows the comparison between the two treatment modalities.

**Table 2:** Comparison of the subjective, radiological, functional, and clinical outcomes between the two groups according to the Honkonen and Jarvinen criteria ORIF - Open Reduction Internal Fixation

VARIABLES		ORIF	Ilizarov	P-VALUE
HJ SUBJECTIVE OUTCOME	EXCELLENT	11 (16.2%)	07 (10.1%)	0.29
	GOOD	36 (52.9%)	41 (59.4%)	0.44
	FAIR	14 (20.6%)	12 (17.4%)	0.63
	POOR	07 (10.3%)	09 (13%)	0.61
HJ CLINICAL OUTCOME	EXCELLENT	10 (14.7%)	11 (15.9%)	0.80
	GOOD	29 (42.6%)	32 (46.4%)	0.60
	FAIR	16 (23.5%)	21 (30.4%)	0.36
	POOR	13 (19.1%)	05 (7.2%)	0.07
HJ FUNCTIONAL OUTCOME	EXCELLENT	12 (17.6%)	15 (21.7%)	0.54
	GOOD	34 (50%)	28 (40.6%)	0.26
	FAIR	12 (17.6%)	21 (30.4%)	0.08
	POOR	10 (14.7%)	05 (7.2%)	0.16
HJ RADIOLOGICAL OUTCOME	EXCELLENT	06 (8.8%)	08 (11.6%)	0.59
	GOOD	33 (48.5%)	35 (50.7%)	0.79
	FAIR	20 (29.4%)	19 (27.5%)	0.80
	POOR	09 (13.2%)	07 (10.1%)	0.57

**Figure 2:**
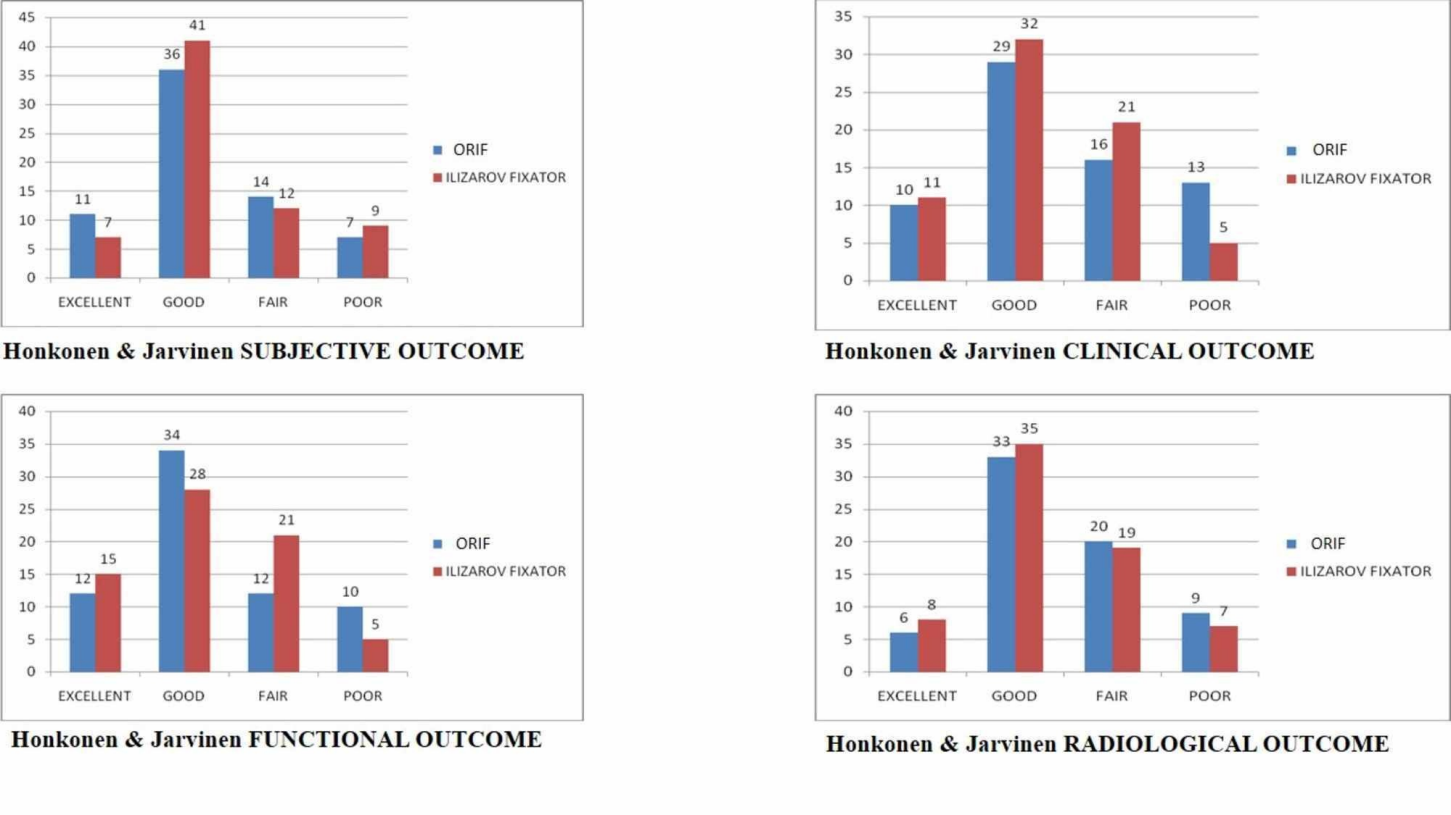
Bar charts comparing the subjective, functional, clinical and radiological outcomes

## Discussion

Our results suggest that there was no significant difference in the subjective, functional, clinical, and radiological outcomes between the patients treated by internal fixation versus external fixation. Therefore, we were unable to establish which treatment modality was better than the other. One of the reasons that there was no significant difference was because the surgery was performed by senior consultants who had vast experience and advanced training in treating complex trauma and fractures, respectively.

When citing previous literature on the management of Schatzker VI and VI fractures, we found limited publications comparing ORIF with external fixation [17]. Most of the publications focussed on either of the treatment methods. Table 3 shows some of the published data that compares both forms and treatment and provides a conclusion.

**Table 3:** Illustrates previous literature between plating and external fixator Ex-Fix - External Fixation, ORIF - Open Reduction and Internal Fixation, RCT - Randomized Control Trial, USA - United States of America, UK - United Kingdom

Author (year)	Design	Country of Origin	Sample Size	Intervention	Inference
Canadian Orthopaedic Trauma Society [12]. (2006) Hall et al [15]. (2009)	RCT	Canada	82	Ex-Fix 43 ORIF 40	There were no differences in the functional results and quality of reduction between ORIF and the Ilizarov group at two years. Only a few patients were able to return to their normal pre‑injury activities. Regardless of the treatment method, patients with this injury have significant residual limb-specific and general-health deficits at two years of follow-up.
Mallik et al [18]. (1992)	Non-RCT	USA	17	Ex-Fix-10 ORIF- 7	There was no difference in outcome between the osteosynthesis group and the Ilizarov group.
Metcalfe et al [19]. (2015)	Metanalysis	USA	419	Ex-Fix-220 ORIF- 209	External ring fixators and plating both are acceptable treatment modalities.
Zhao et al [20]. (2017)	Metanalysis	China	519	Ex-Fix- 239 ORIF- 280	Although external fixation may offer some advantages, both were acceptable strategies in managing complex tibial plateau fractures
Chan et al [17]. (2012)	Non-RCT	UK	58	Ex-Fix- 36 ORIF- 25	There was no significant difference In the treatment outcome between bicondylar tibial plateau Fractures treated with either method
Yu et al [21]. (2015)	Metanalysis	China	885	Ex-Fix- 561 ORIF- 331	There was no significant difference in the functional and radiological outcomes.
Krupp et al [22].	Non-RCT	USA	58	Ex-Fix- 30 ORIF- 28	When compared with external fixation, locked plating was associated with an increased rate of fracture union, a decreased incidence of articular malunion, a better range of knee motion, and decreased overall complications.

Pun et al. obtained a mean of 128.1 degrees of posttreatment knee flexion in his series of 22 Schatzker type V and VI fractures; the highest reported in the current literature [23]. The average results of our study were 115.51 degrees with 119.26 degrees in the Ilizarov-treated group and 111.71 degrees in the ORIF group. Better results in Pun et al. cohorts could be due to smaller sample sizes as compared to ours. Furthermore, his study does not compare the results of plating and Ilizarov separately.

However, our results were comparable with Canadian Orthopaedic Trauma Society, which showed that there was no statistical significance in the range of motion at two years (p-value: 0.09) with an average 109 degrees in the ORIF group as compared to 120 degrees in Ilizarov-treated patients [12].

A recent Cochrane review by McNamara et al. evaluating four randomized and two quasi-randomized trials, comparing ORIF with Ilizarov, did not find enough evidence to ascertain the best method of fixation. Current evidence does not contradict the idea of the best results obtained when using limited exposures to treat these fractures [24].

Our findings are also consistent with the 2015 Cochrane Review, as both groups showed overall good results and the final decision of the treatment modality depends on the surgeon's expertise and practice and the ability to overcome or resolve the complications secondary to the treatment offered.

The limitation of the study was that it was a single-center study, and the weakness of the study was its retrospective nature. The strengths of the study were the large sample size in both the groups and follow-up to a minimum of two years.

Although the small population size, the lack of control groups, and the various functional assessment tools in the majority of the past publications limit the strength of any recommendations that could be made regarding the optimal options of the surgical method in bicondylar fractures, which makes our study relevant to current practice.

## Conclusions

In conclusion, no single fixation strategy is superior in all cases, and the decision to optimally treat these complex injuries varies on a case-by-case basis. Above all, the final decision depends on the surgeon's expertise and practice and the ability to overcome and resolve the complications secondary to the treatment offered.
